# Public health challenges: A new scientific path and platform

**DOI:** 10.1002/puh2.5

**Published:** 2022-03-16

**Authors:** Don Eliseo Lucero‐Prisno

**Affiliations:** ^1^ Department of Global Health and Development London School of Hygiene and Tropical Medicine London UK; ^2^ Harvard Medical School Harvard University Boston Massachusetts USA; ^3^ Faculty of Public Health Mahidol University Bangkok Thailand

The COVID‐19 pandemic will likely come down in history as pivotal in the trajectory of the research and scientific community. It opened the gate to a deluge of scientific and societal challenges and opportunities that paved the way to many changes in the way we work and interact in the global scientific world. The enormity and magnitude of the pandemic presented numerous challenges, and brought the scientific community into unchartered waters. As COVID‐19 started to spread globally, so did the unabated production of research and publications [1]. Suddenly, the lockdowns forced many scientists and researchers, including the novices, to sit down at home and fill their time with writing papers—a much‐needed opportune forced upon them by the situation. For a number of them, there was no more complaint for lack of time and concentration. Researchers were able to work on their untouched data. Scientists had more time to rethink and revise their theories. Research enthusiasts tried their hand in writing. Online communication technology paved the way to the overnight creation of many research groups. This resulted in the unprecedented production of a large number of manuscripts.

One opportunity led to another: the need created a ripe opportunity for publishers to establish new journals. The industry reacted to the market opportunity. New publishers also started to sprout. Suddenly, one's email is flooded with requests for manuscripts and reviews from both new and established scientific journals. With the overproduction of papers also came the overproduction of information. The scientific community became a contributor to the phenomenon of the *infodemic* [2]. The rush of manuscripts to be published by enterprising new publishers that took advantage of a new market opportunity sometimes bypassed the critical and meticulous process of scientific journal publishing. Rules of peer‐review and scientific scrutiny were not anymore properly observed. The production of unsound scientific findings added to the misinformation which the unknowing public consumed and shared through a highly dynamic social media. In today's era of *fake news*, where society is continuously flooded with information, the scientific community suddenly got challenged to be more vigilant and to promote high‐quality science.

It is in this context that the *Journal of Public Health Challenges* was conceived. As the inaugural Editor‐in‐Chief of *Public Health Challenges*, I am privileged and challenged to steer this journal to the scientific level that a scientific journal should have. It is a response to these trying times when many unprecedented public health challenges suddenly transpired. I feel lucky that the journal title, *Public Health Challenges*, encapsulates a continuing human saga. In whatever time we are embedded in, there are always predicaments to health within the public space. Thus, the first major role of the journal is to be a platform to showcase and present latest and relevant scientific findings in public health that will impact the global population. This has always been how scientific journals are seen. There are, however, additional functions and roles that this journal is envisioned to play. *Public Health Challenges* is meant to provide a venue for discussion and debate. Critical public health should be an integral approach in looking at human health [3]. A healthy societal debate in a civilized, if not a scientific manner, should be one of the approaches in the way we move forward in this highly globalized and interconnected world. This is what the journal will encourage—to provide voices to all sectors of society.

Despite the thousands of scientific journals in health and medicine, it is astonishing to note that based on journal search, the *Web of Science* and *Scopus* websites yield only a combined total of a little bit more than 200 open access journals in 2021 under the category *public, environmental and occupational health*. This makes less than a hundred scientific journals, purely in public health, to be considered to be of very high quality. Using *Web of Science* and *Scopus* as the benchmark of quality by virtue of the number it indexes, it is quite telling of the scientific and editorial quality of scientific journals nowadays. With the proliferation of thousands of new journals launched during the pandemic, it just made matters worse. This is what this new journal will uphold—to keep a very high scientific and ethical standard and lead the way for other journals. Thus said, there is not enough space for many good manuscripts to be accommodated in high quality journals. Good scientists and researchers deserve good journals, while the need for good journals is dire.

I envision this journal to be innovative and trail‐blazing. It should be a venue for celebration. A manuscript submitted by researchers is a product of scientific imagination and hard work. The mere fact that a manuscript is submitted is a cause for celebration. The journal will share this triumph by providing the avenues to highlight one's research findings once published. Innovative technology will be tapped to promote ones work.

Another innovation of *Public Health Challenges* is to perform what a modern journal does—lead the health research agenda. A journal should not merely be a passive platform, but one that leads and influences. It will identify trends and lead directions. It will push for more research for emerging public health areas that need urgent attention. It will be an authority to compel the community to carry out research on pressing areas. After all, evidence‐based policy and practice are central in the dynamic functioning of our society.

Journals have emerging roles, if you noticed. They have an archival role; they archive information that may be used by future generations, and that may be relevant in future public health challenges. Had information of previous pandemics been archived meticulously, we could have used such information to address the current pandemic. Thus said, no publication comes to waste. The criticism that research carried out simply for the sake of research is not a valid argument anymore. Studies produced will always have utility, either in the present or in the future. Research is now seen as the use of existing knowledge in a new and creative way, so as to generate new concepts, methodologies and insights. Previous findings can be synthesised and analysed, leading to new and creative outcomes. Nothing is wasted.

This journal advocates to democratize science. Science is a global public good [4]. Everyone deserves to publish, regardless of their geographic location, scientific ability or their ability to pay. *Public Health Challenges* will facilitate an equitable access to publish manuscripts with special emphasis to low‐ and middle‐income countries, least published countries and marginalized scientific communities. This will be accomplished through a number of ways, including a support fund. Information and ideas should not merely be the domain of the academic elite. The evidence that the determinants of health are more pronounced among those who lack many opportunities, including not getting published, is a compelling reason why *Public Health Challenges* is best voiced out by those who experience it. Only through democratization of science will we hear more of the voices of those who are impacted the most—their wants, needs, aspirations and insights.

Young researchers are crucial in the advancement and development of science. They will be provided a special platform in this journal. The journal will provide a training ground for them as they will be the scientific leaders in the future. A *Youth Editorial Board* will be set up. Young researchers, including students, will also be encouraged to publish their work. Initiatives to mentor and nurture young and emerging scientists will be an endeavour of the journal. When the time comes, they will be among those leading the scientific community with more confidence, and with the right frame of thinking.

The journal considers *Editorial Board Members* as partners in achieving the goals mentioned above. They will be an equal partner and not merely passive participants or only called upon as reviewers or manuscript handlers. Their collective expertise will be tapped to steer the journal in all its envisioned goals. The journal will be inclusive. It will embrace and nurture a Board from different persuasions, scientific fields and geographies. It is with diversity that a collective voice becomes strong and powerful. The Board will be given a platform to present stands on pressing global issues and debates.

This journal also provides a personal connection to me. I am a former PhD student of Professor Gareth Williams at Cardiff University in Wales, United Kingdom. For many years, he was the Editor‐in‐Chief of Wiley's successful journal, *Sociology of Health and Illness* [5]. Every week when I had my supervision with him, I was awed by the numerous manuscripts scattered on his table. I always wondered what it is like to be an editor. This started my interest in scientific journal work. Aside from doing my own research, I started to dabble in reviewing, joining editorial boards, handling manuscripts, and guest editing special issues. I always looked up at him as I was amazed of his work. He succumbed to a rare illness during the height of his academic career. Coming to Wiley as Editor‐in‐Chief, just like him, is one way of thanking him for shaping my scientific career and to honour our good memories of him.

## AUTHOR CONTRIBUTIONS

Don‐Eliseo Lucero‐Prisno III; Conceptualization, Formal analysis, Methodology.

## CONFLICT OF INTEREST

The author declares no competing interests. The author is the Editor‐in‐Chief of *Public Health Challenges*. The manuscript was independently reviewed.

## ETHICS STATEMENT

This is an editorial. There is no need for ethical approval.

## Data Availability

No database or primary data was used in preparing the manuscript.
